# The Effect of Exercise on Salivary Viscosity

**DOI:** 10.3390/diagnostics6040040

**Published:** 2016-11-16

**Authors:** Antoon J. M. Ligtenberg, Erwin H. S. Liem, Henk S. Brand, Enno C. I. Veerman

**Affiliations:** Department of Oral Biochemistry, Academic Centre for Dentistry Amsterdam, 1081LA, Amsterdam, The Netherlands; Erwinliem@hotmail.com (E.H.S.L.); h.brand@acta.nl (H.S.B.); e.veerman@acta.nl (E.C.I.V.)

**Keywords:** exercise, MUC5B, amylase, protein

## Abstract

A common experience after exercise is the presence of a thick and sticky saliva layer on the oral surfaces, which causes a feeling of a dry mouth. Since the salivary mucin MUC5B is responsible for the visco-elastic behavior of saliva, in the present study we explored the effect of exercise on both the salivary viscosity and the secretion of MUC5B in saliva. Twenty healthy dental students performed an aerobic exercise by cycling for 15 min on cycle-ergometers at a heart rate of 130–140 beats per minute. Saliva was collected at three time points: before exercise, immediately after exercise and after 30 min recovery. Salivary flow rate, viscosity, amylase activity, total protein, carbohydrate and MUC5B concentration were determined. Salivary flow rate, protein and amylase did not change significantly. Immediately after exercise, the salivary viscosity and carbohydrate concentration were significantly higher than at baseline and after 30 min recovery. Immediately after exercise, the MUC5B concentration was significantly higher than after 30 min recovery. It is concluded that the presence of thick saliva after exercise is at least partially due to an increased secretion of MUC5B.

## 1. Introduction

Strenuous exercise has been associated with a reduced immune function and an increased susceptibility to infections, particularly of the upper respiratory tract (URTI) [1,2,3,4,5]. Exercise-related URTIs are associated with a decreased immune function at mucosal surfaces and a decreased secretion rate of salivary immune proteins [2]. Therefore, saliva has been used as a marker for mucosal immunity [6,7]. Saliva plays an important role in the body’s first defense to ingested pathogens. It covers the hard and soft tissues in the oral cavity, forming a complex barrier that is important for hydration, lubrication, pathogen exclusion and resistance to proteolytic digestion. The high-molecular-weight mucin MUC5B comprises the matrix of this complex network and forms complexes with other innate immune components in saliva, including cystatins, histatins, immunoglobulins, proline-rich proteins and MUC7 [8,9].

Physical exercise has different effects on salivary parameters. After short, intense exercise, secretion of secretory IgA (s-IgA) decreased, whereas total protein increased [6]. With an exercise intensity above the anaerobic threshold, the secretion of total protein, amylase, lysozyme, lactoferrin, chromogranin A and MUC5B increased [10,11,12,13,14]. The increased secretion of proteins and immune components may be explained by the neuronal regulation of saliva secretion [15]. The salivary glands are both sympathetically and parasympathetically innervated. Parasympathetic stimulation elicits a high volume of watery saliva that is low in protein content. Saliva elicited by sympathetic stimulation is low in volume and high in protein content, mainly due to increased exocytosis of salivary proteins from salivary cells. The presence of elevated levels of salivary amylase is considered an indicator of increased adrenergic activity [16,17]. Since exercise is associated with enhanced sympathetic nervous system activation, it seems logical to assume that exercise will increase the secretion of several innate immune proteins.

After exercise, the salivary viscosity is increased, which may have several causes [18]. For example, dehydration with a concomitant decreased secretion rate of saliva may result in increased concentrations of proteins and mucins [19]. In addition, mouth breathing during exercise may lead to increased evaporation of water and subsequent thickening of saliva. Increased viscosity might also be due to the increased secretion rate of MUC5B, similar to the increased secretion of other proteins after exercise. A previous paper demonstrated that MUC5B secretion increased after intense exercise [14]. This was somewhat surprising, because MUC5B secretion is thought to be mediated by parasympathetic stimulation and intense exercise is a strong sympathetic stimulus [15].

In the present study we investigated the effect of exercise on saliva viscosity and the salivary mucin MUC5B. To exclude the potential effects of excessive mouth breathing and considerable changes in saliva above the anaerobic threshold, which may affect the viscosity of saliva, participants performed an aerobic exercise with moderate intensity. Participants exercised for 15 min on a cycle ergometer with a heart rate between 130–140 beats per minute [20], and the effects on the saliva secretion rate, viscosity, protein, carbohydrate, amylase and MUC5B were determined.

## 2. Materials and Methods

### 2.1. Participants

This study was approved by the Medical Ethical Committee of the VU Medical Center Amsterdam in accordance with OB13-453. Twenty healthy dental students of the Academic Centre for Dentistry in Amsterdam were recruited, 12 men and eight women aged 18–32 years (mean age = 25 years). They were non-smokers and performed physical exercise at least two times a week. They were instructed beforehand about the research design and gave written informed consent.

### 2.2. Experimental Procedure

The subjects exercised on a cycle-ergometer with handgrip heart rate monitor (Life Fitness upright lifecycle 95C, T-Fitness, Amsterdam, The Netherlands) between 9:30 a.m. and 11:30 a.m. in a conditioned environment of 21.6 °C. They were instructed to refrain from doing strenuous physical activities 24 h before the test and not to eat or drink anything 1 h before the experiment. Each subject cycled for 15 min and was instructed to keep the heart rate between 130–140 beats per minute which was below the anaerobic threshold [20]. Unstimulated saliva was collected at three time points: before exercise, immediately after exercise and after a 30 min recovery period.

### 2.3. Saliva Collection and Analysis

Unstimulated saliva was collected by means of the spitting method [21]. Before collection, each participant was instructed not to breathe through the mouth or to talk during the collection of saliva. After swallowing all the saliva in the mouth, saliva was collected for 5 min. Each participant expectorated in a disposable container every 30 s. The samples were immediately put on ice after collection.

The volume of saliva was determined by weighing, assuming a density of 1.0 g/mL for saliva. pH was measured with a pH meter (PHM240 pH/Ion Meter, Radiometer BV, Zoetermeer, The Netherlands).

### 2.4. Viscosity Measurement

Viscosity was measured with freshly collected saliva. Before viscosity measurements saliva was centrifuged (1 min, 10,000× *g*) to remove cellular debris. The viscosity of the cleared saliva was measured with a viscometer (Vilastic 3, Vilastic Scientific Inc., Austin, TX, USA), as described previously [22]. The viscometer was calibrated before every measurement with demineralized water. Between measurements the tubings of the viscometer were flushed with demineralized water.

### 2.5. Saliva Analysis

Freshly collected saliva was diluted 1:1 with 150 mM NaCl and homogenized for 1 min with a Vortex mixer (Vortex-Genie, Wilten BV, Etten-Leur, The Netherlands). The dilution lowered viscosity, thus facilitating centrifugation and sample manipulation, and prevented protein aggregation and precipitation during saliva freezing and storage. Then saliva was clarified by centrifugation for 5 min at 10,000× *g* at room temperature. The cleared supernatants were stored at –20 °C until use.

Protein concentration was determined using the Bicinchoninic Acid (BCA) protein assay (Pierce, Rockford, IL, USA) [23]. The amylase activity was measured with the EnzCheck Ultra Amylase Assay Kit (Thermo Scientific, Paisley, UK), using a fluorogenic starch substrate (DQ™) [23]. MUC5B concentration was determined as described previously with an anti sulfo-Lewis^a^ monoclonal antibody F2 [23]. MUC5B concentration was expressed as units/mL. Unstimulated saliva of one person was used as a standard of one unit. Carbohydrate concentration was measured according to the method of Kilcoyne et al. [24]. 25 µL saliva was mixed with 75 µL 0.1% sodium meta periodate in 10% acetic acid in non-affinity microplates (Greiner) and incubated for 2 h at 37 °C. Thereafter, 100 µL Schiff’s reagent (Merck, Darmstadt, Germany) was added. After incubation for 1 h at 37 °C color development was measured at 570 nm with a microplatereader (Thermo Scientific, West Palm Beach, FL, USA). *N*-acetylgalactosamine (0–2 mg/mL) was used as a standard.

### 2.6. Statistical Analysis

The data collected at different time points were statistically compared by using the Friedman test for multiple related samples, using the Wilcoxon test as *post-hoc* procedure to compare the individual time points. IBM SPSS Statistics 21 (IBM, Armonk, NY, USA) for Windows was used for analyzing the data. Significance level was set at *p* < 0.05.

## 3. Results

The effects of exercise on various salivary parameters are presented in Table 1. The salivary parameters that showed significant changes using the Friedman test for multiple related samples were subsequently compared pairwise with the Wilcoxon test. These results are shown in Table 2.

No significant differences were found in the pH and saliva flow rate. The protein concentration and secretion rate as well as the amylase concentration and secretion did not significantly change. The viscosity of saliva significantly increased immediately after exercise (*p* = 0.04) and decreased to sub-baseline values after 30 min recovery. Immediately after exercise, the MUC5B concentration and secretion rate were significantly higher than after 30 min recovery. The MUC5B concentration per mg protein was significantly decreased after 30 min recovery.

## 4. Discussion

An increase in salivary viscosity after exercise has been suggested before [18]. However, to our knowledge, this study is the first to demonstrate a significant increase in salivary viscosity directly after exercise. Since the salivary flow rate did not decrease during exercise, thickening of saliva due to mouth breathing or dehydration could not explain the observed increase in viscosity. Since MUC5B is the main determinant of salivary viscosity, we suggest that the increase in the MUC5B concentration causes the increase in salivary viscosity.

In previous studies, clear changes in the composition of saliva were reported when subjects exercised above the anaerobic threshold. Above the anaerobic threshold, amylase [25,26], total protein [10], lactoferrin [27], chromogranin A [12], lactate [28], sodium and chloride all increased [6,29]. The present finding that exercise had no effect on the protein concentration is possibly due to the fact that the subjects exercised at a low to moderate intensity, well below the anaerobic threshold.

The saliva secretion rate is regulated by the synergistic actions of the parasympathetic and sympathetic nerves of the autonomic nervous system. The secretion of amylase and other proteins is evoked primarily by sympathetic stimulation. Mucin secretion has been suggested to be evoked primarily by parasympathetic stimulation of the sublingual and minor glands [15]. However, our study showed an increased MUC5B secretion rate immediately after moderate exercise. This increase was not significant, but in a previous study we showed a significant increase in MUC5B secretion immediately after moderate exercise [14]. Possibly, the cold air that is inhaled by mouth breathing during exercise stimulates mucus secretion by the minor glands in a similar way as in the lung epithelium [30]. This effect may have been stronger in the previous study where subjects exercised outdoors in colder circumstances (7–15 °C) than in the present study (21.6 °C).

## 5. Conclusions

In conclusion, this study shows that there is a temporary increase in the viscosity of saliva immediately after moderate exercise, which is probably caused by an increase of the MUC5B secretion rate.

## Figures and Tables

**Table 1:** Effect of exercise on the saliva flow rate, pH, viscosity and composition of saliva. Values are mean ± standard deviation.

Time Point	Before Exercise	Immediately after Exercise	After 30 Min Recovery
Saliva flow rate (mL/min)	0.56 ± 0.21	0.58 ± 0.20	0.64 ± 0.30
pH	7.31 ± 0.39	7.32 ± 0.44	7.31 ± 0.33
Viscosity (mP/s) *	3.19 ± 3.39	3.86 ± 3.78	2.23 ± 1.45
Protein concentration (mg/mL)	1.18 ± 0.41	1.20 ± 0.50	1.15 ± 0.39
Protein secretion rate (mg/min)	0.63 ± 0.26	0.65 ± 0.19	0.71 ± 0.36
MUC5B concentration (units/mL) *	1.28 ± 0.94	1.47 ± 1.15	0.92 ± 0.68
MUC5B secretion rate (units/min) *	0.64 ± 0.46	0.75 ± 0.49	0.55 ± 0.42
MUC5B/protein (units/mg) *	1.05 ± 0.66	1.16 ± 0.64	0.81 ± 0.55
Carbohydrate concentration (mg/mL) *	1.23 ± 0.40	1.34 ± 0.42	1.21 ± 0.51
Carbohydrate secretion rate (mg/min)	0.65 ± 0.18	0.76 ± 0.24	0.76 ± 0.39
Carbohydrate/protein (mg/mg)	1.19 ± 0.54	1.27 ± 0.41	1.18 ± 0.53
Amylase concentration (units/mL)	128 ± 74	140 ± 77	128 ± 88
Amylase secretion rate (units/min)	73 ± 55	80 ± 49	83 ± 70
Amylase/protein (units/mg)	105 ± 43	120 ± 56	106 ± 57

* *p* < 0.05.

**Table 2:** Effect of exercise on the composition of saliva: comparison of different time points. Parameters that showed significant changes according to the overall comparison with Friedman test were *post-hoc* pairwise compared with the Wilcoxon test.

Overall Comparison		Time Points Compared		
		1–2	1–3	2–3
Viscosity (mP/s)	0.011 *	0.040 *	0.370	0.006 *
MUC5B concentration (units/mL)	0.001 *	0.104	0.008 *	0.001 *
MUC5B secretion rate (units/min)	0.026 *	0.117	0.191	0.008 *
MUC5B/mg protein (units/mg)	0.003 *	0.086	0.030 *	0.007 *
Carbohydrate concentration (mg/mL)	0.006 *	0.036	0.398	0.022 *

Time points: 1 = before exercise; 2 = immediately after exercise; 3 = after 30 min recovery * *p* < 0.05.
